# Anatomical Variants of the Uncinate Process: A Challenge in the Endoscopic Surgery of the Nose and Paranasal Sinuses

**DOI:** 10.7759/cureus.50914

**Published:** 2023-12-21

**Authors:** Mariana Fikir, Jose L Treviño-González, Baltazar González Andrade, Josefina Alejandra Morales Del Angel

**Affiliations:** 1 Otolaryngology - Head and Neck Surgery, Hospital Universitario Dr. José Eleuterio González, Monterrey, MEX

**Keywords:** computed tomography, paranasal sinuses, sinus surgery, anatomic variation, uncinate process

## Abstract

Importance

To identify the anatomical variants of the uncinate process relevant to surgical intervention during the nose and paranasal sinus surgeries.

Objective

To evaluate the frequency of anatomical variants of the uncinate process in a population of northeast Mexico and compare it with another population.

Methods

Retrospective study, descriptive and analytical, randomly selected patients with radiological evaluation at Hospital Universitario Dr. José Eleuterio González, Monterrey, Mexico. Images were obtained from the Radiology Department. A total of 149 patients aged from 18 to 79 years with paranasal sinus-CT performed between January 2019 and December 2021 were analyzed. The variables evaluated were uncinate process anatomical variations by age group, radiological classification of the superior attachment of the uncinate process, and morphological variations.

Main outcomes and measures

The primary study outcome was the determination of the most frequent insertion of uncinate process in the northeast Mexican population.

Results

The 149 CT scans comprised 71 females with a mean age of 38.28 ± 16.7 years and 78 males, with a mean age of 41.8 ± 15.01 years. The most frequent uncinate process of superior attachment was type one, observed in 57.7% of males (n=45) and 50.7% of females (n=37) (p=0.494). Type one was most observed on the right side (57.7%). Type four was the second most common type, present in 12.8% of males (n=10) and 12.7% of females (n=9) (p=0.82).

Conclusion

Knowledge about the types of variations in the insertion of the uncinate process is fundamental prior to any endoscopic sinus surgery. The surgeon must be familiar with this detail when approaching patients with sinonasal pathology.

## Introduction

The uncinate process (UP) of the ethmoid bone, part of the osteomeatal complex (OMC), with a curved-sickle shape that arises from the anteroinferior area of the ethmoid labyrinth just behind the lacrimal bone is an important landmark during endoscopic sinus surgery [1,2]. The resection of the UP provides the visualization of the maxillary antrum, one of the crucial steps during functional endoscopic sinus surgery procedures [3]. Superiorly, the UP may have distinct types of insertion, which alters the drainage passage of the frontal sinus. Landsberg et al. proposed a classification of the superior insertion of the UP regarding its insertion into the lamina papyracea, the posterior wall of the agger nasi cell, the junction of the middle turbinate with the cribriform plate, skull base, and the middle turbinate [4].

The UP has multiple anatomical variants classified according to Stammberger [5]: (1) superior insertion, type one: the UP inserts into the lamina papyraceous, type two: extension superior to the base of the skull, type three: middle turbinate insertion; (2) medial insertion; (3) side insert; and (4) pneumatized UP.

Landsberg et al. [4] emphasized the position of the superior attachment of UP for adequate exposure of the frontal sinus and appropriate dissection during frontal recess surgery. They described a radiological classification of six different types of UP according to the position of its superior attachment utilizing CT for its evaluation.

Stammberger et al., described distinct classifications regarding the shape, attachment, hypertrophy, and pneumatization of the UP [5]. Previous studies have evaluated the role of UP classification in frontal sinus sinusitis, chronic rhinosinusitis, and frontal mucoceles, yielding controversial results [2,6,7]. 

In Mexico, there are few research articles that talk about the anatomical variants of UP insertion. We found one in the search that deals with this specific topic, which was carried out in a tertiary care unit whose analysis was compared with that of another study observing relevant data regarding ethnic groups. Anatomical variations of the shape and superior attachment of the UP vary individually and among populations.

CT has served as the gold standard for OMC evaluation and has become the single most important study for the preoperative evaluation of this structure [8]. The preoperative assessment of anatomical variations is mandatory to provide an adequate surgical plan and avoid intraoperative complications. With the above in mind, we evaluated the CT of northeastern Mexican patients to analyze the most common types of classification of the UP in our population and to assess differences between gender and age groups.

## Materials and methods

Study design

A retrospective, descriptive, and analytical study of randomly selected patients with radiological evaluation was conducted at the university hospital "Dr. José Eleuterio Gonzalez," a tertiary care unit. The research protocol OT22-00004 was approved by the local Research and Institutional Ethics Committee of Hospital Universitario Dr. José Eleuterio González. The authors assert that all procedures contributing to this work comply with the ethical standards of the relevant institutional guidelines on human experimentation and the Helsinki Declaration of 1975, as revised in 2008.

Subjects

A consecutive sample of 149 patients aged from 18 to 79 years with paranasal sinus-CT (PNSCT) was performed between January 2021 and December 2021. Patients with a previous history of chronic rhinosinusitis, nasal polyposis, sinonasal diseases or tumors, skull base surgery or pathology, previous endoscopic sinus surgery, nasal trauma, facial dysmorphisms, or nasal trauma were excluded from the study.

Image acquisition

Images were obtained at a tertiary care unit hospital "Dr. José Eleuterio Gonzalez" at the Radiology Department. Images were gathered using a 64-slice CT (GE Medical Systems light speed VCT, Waukesha, WI, EUA) with a voltage of 120 kV, effective mAs of 18, and a field of view of 142x278 mm. Obtained images contained 1.25 mm axial slices and coronal and sagittal reconstructions in a high-definition bone window. Images were evaluated by head and neck radiologists.

Studied variables

The following variables were evaluated: UP anatomical variations by age group. Age was divided into six groups: group one (18-29 years), group two (30-39 years), group three (40-49 years), group four (50-59 years), group five (60-69 years), and group six (>70 years). 

**Figure 1:**
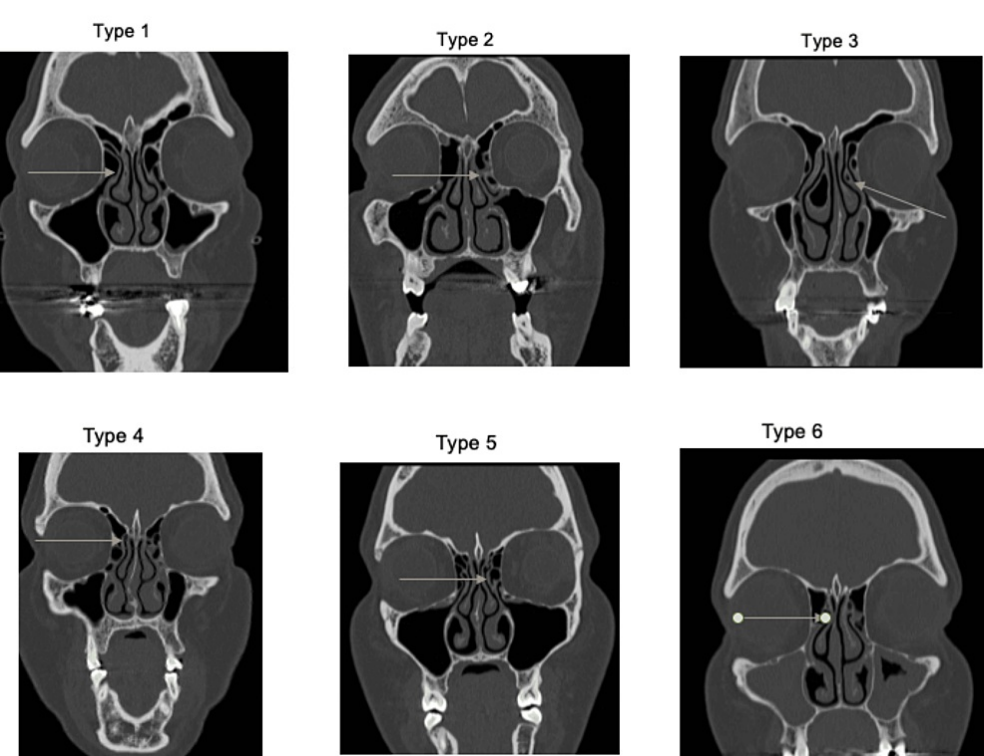
Different types of insertion of the uncinate process Source: Landsberg et al. [4] Type one: Insertion to the lamina papyracea Type two: Insertion to the posterior wall of the agger nasi cell Type three: Insertion to the lamina papyracea and union of the middle turbinate with the cribriform plate Type four: Insertion at the union of the middle turbinate with the cribriform plate Type five: Insertion to the ethmoidal skull base Type six: Insertion at the middle turbinate

Radiological classification of the superior attachment of the UP was performed according to Landsberg et al. [4]. Type one UP is inserted into the lamina papyracea. Type two inserts in the posteromedial wall of the agger nasi cell. Type three is to both the lamina papyracea and the junction of the middle turbinate with the cribriform plate. Type four UP is inserted into the union of the middle turbinate with the cribriform plate of the ethmoid bone. Type five is inserted into the skull base. Type six inserts into the middle turbinate (Figure 1).

Morphological variations of the uncinate process were classified according to Güngör et. al in atelectatic, curved, bifid, deviated, and uncinate bulla [9].

Statistical analysis 

Statistical analysis was performed using Statistical Product and Service Solutions (SPSS, V25.0) (IBM SPSS Statistics for Windows, Armonk, NY). Categorical variables are reported as percentages and frequencies using Pearson's x2 test; continuous variables are reported as means and standard deviations using Fisher's exact test for 2x2 tables. An unpaired Student’s t-test or Mann-Whitney U test was used to compare continuous variables. P<0.05 was considered statistically significant.

## Results

We evaluated 149 CT scans, with females with a mean age of 38.28 ± 16.7 years, and males with a mean age of 41.8 ± 15.01 years. Overall, 47.7% of patients were females (n=71) (Table 1).

**Table 1:** Distribution of men and women and types of insertion classifications of the uncinate process Pearson correlation coefficient test

Variables	Male	Female	P value
	n = 78	n = 71	
Age, mean (SD)	41.8 ± 15.01	38.28 ± 16.7	0.249
Right UP classification			
Type 1, n (%)	45 (57.7)	37 (50.7)	0.494
Type 2, n (%)	4 (5.1)	8 (11.3)	0.294
Type 3, n (%)	4 (5.1)	13 (18.3)	0.026
Type 4, n (%)	10 (12.8)	9 (12.7)	0.821
Type 5, n (%)	5 (15.4)	4 (5.6)	0.072
Type 6, n (%)	6 (3.8)	1 (1.4)	0.177
Left UP classification			
Type 1, n (%)	43 (55.1)	33 (46.5)	0.449
Type 2, n (%)	8 (10.3)	9 (12.7)	0.959
Type 3, n (%)	3 (6.4)	3 (16.9)	0.347
Type 4, n (%)	4 (6.4)	9 (12.7)	0.041
Type 5, n (%)	5 (14.1)	5 (9.9)	0.195
Type 6, n (%)	6 (3.8)	1 (1.4)	0.373

UP anatomical variations by sex

The most frequent UP superior attachment was type one, observed in 57.7% of males (n=45) and 50.7% of females (n=37) (p=0.494). Type one UP was most commonly observed on the right side (57.7%). Type four was the second most common type of UP, present in 12.8% of males (n=10) and 12.7% of females (n=9) (p=0.82). No significant differences in the type of superior insertion of UP and laterality were observed between males and females (Table 1).

UP anatomical variations by age group

The most common age group was from 18 to 29 years (n=54) with a mean age of 23.8 ± 3.3. Among all age groups, type one UP was the most common classification observed. A curved UP was the most common variation present in our population in all age groups, followed by uncinate bulla. No statistical differences were observed in UP classification and variations between groups (Table 2).

**Table 2:** Distribution of insertion types according to each age group Pearson correlation coefficient test

Age group	18-29 (%)	30-39	40-49	50-59	60-69	>70	P value
	n = 54	n = 26	n = 23	n = 23	n = 18	n = 5	
Age, mean (SD)	23.8 ± 3.3	34.5 ± 3.8	44.6 ± 2.9	55.3 ± 3.3	63.1 ± 3.1	73.2 ± 1.6	0.5
Female, n (%)	32 (45.1)	11 (15.5)	8 (11.3)	10 (14.1)	6 (8.5)	4 (5.6)	0.132
Right-sided UP classification							
Type 1, n (%)	28 (51.9)	11 (42.3)	15 (65.2)	14 (60.9)	2 (40)	2 (40)	0.461
Type 2, n (%)	4 (7.4)	2 (7.7)	2 (8.7)	0 (0)	1 (20)	1 (20)	0.483
Type 3, n (%)	8 (14.8)	4 (15.4)	0 (0)	1 (4.3)	1 (20)	1 (20)	0.453
Type 4, n (%)	7 (13)	6 (23.1)	2 (8.7)	3 (13)	0 (0)	0 (0)	0.639
Type 5, n (%)	4 (7.4)	3 (11.5)	4 (17.4)	4 (17.4)	1 (20)	1 (20)	0.534
Type 6, n (%)	3 (5.6)	0 (0)	0 (0)	1 (4.3)	0 (0)	0 (0)	0.926
Left UP classification							
Type 1, n (%)	26 (48.1)	14 (53.8)	9 (39.1)	12 (52.2)	12 (66.7)	3 (60)	0.719
Type 2, n (%)	8 (14.8)	3 (11.5)	3 (13)	1 (4.3)	1 (5.6)	1 (20)	0.732
Type 3, n (%)	9 (16.7)	4 (15.4)	3 (13)	1 (4.3)	3 (16.7)	0 (0)	0.873
Type 4, n (%)	5 (9.3)	1 (3.8)	1 (4.3)	5 (21.7)	1 (5.6)	1 (20)	0.433
Type 5, n (%)	4 (7.4)	2 (7.7)	7 (30.4)	4 (17.4)	1 (5.6)	0 (0)	0.043
Type 6, n (%)	2 (3.7)	2 (7.7)	0 (0)	0 (0)	0 (0)	0	0.777

Morphological variations of the UP 

The most observed anatomical variation was a curved uncinate process of 50.4% in males and 49.6% in females, followed by the uncinate bulla present in 66.7% in males and 33.3% in females. No other UP variations were observed in our population (Table 3).

**Table 3:** Morphological variants of the uncinate process, average Pearson correlation coefficient test

UP variants	Male	Female	P value
Atelectatic, n (%)	0 (0)	0 (0)	
Curved, n (%)	59 (50.4)	58 (49.6)	
Bifid, n (%)	1 (25)	3 (75)	
Deviation of the UP tip, n (%)	0 (0)	0 (0)	
Uncinate bulla, n (%)	8 (66.7)	4 (33.3)	
Agger nasi, n (%)	2 (50)	2 (50)	0.924
Right, n (%)	1 (50)	1 (50)	0.947
Left, n (%)	2 (66.7)	1 (33.3)	0.616
UP = uncinate process			

## Discussion

Anatomical variants of the OMC have been the target of studies over time because of the advent of endoscopic surgery of the nose and paranasal sinuses. This study evaluated the most common types and variations of the UP in the Mexican population, being type one UP and curved UP being the most commonly observed anatomic variations by gender and age groups.

As uncinectomy is key during endoscopic sinus surgeries, a preoperative assessment of the OMC anatomy and superior attachment of the UP is crucial. Inadequate evaluation of these variations can result in the failure of the procedure, injury to the lacrimal bone, orbital damage with possible visual disturbances, and skull base injury, among others [10,11].

Previous studies have evaluated important CT landmarks for endoscopic sinus surgery. Vaid et al. proposed a checklist with surgically relevant sinonasal details including the nasal septum, middle turbinate, uncinate process, and OMC [12].

In our study, the most frequent UP superior attachment was type one, observed in 57.7% of males and 50.7% of females (p=0.494). Type one UP was most observed on the right side (57.7%). Type four was the second most common type of UP, present in 12.8% of males (n=10) and 12.7% of females (n=9) (p=0.82). No significant differences in the type of superior insertion of UP and laterality were observed between males and females. The most commonly observed anatomical variation was a curved uncinate process with 50.4% in males and 49.6% in females, followed by the uncinate bulla present in 66.7% in males and 33.3% in females. No other UP variations were observed in our population. This is a morphological variable that is not studied in all studies, as it is with its superior insertion, and it is overlooked that its morphology is also a factor to consider in the approach.

In a study conducted in Mexico City (central Mexico), 139 patients underwent CT in coronal slices. The insertion of the uncinate process that predominated in the study in both genders was the middle turbinate with 68 cases (37.7%), with predominance of the left side (52.9%), and 32 cases (47.5%) being on the right side. The second most frequent insertion was the papyraceous lamina, which was present in 59 cases (32.7%) and was found more frequently on the right side (52.9%). The third insertion in frequency was the skull base with 12 cases (6.67%), being more frequent on the right side (58.33%). The age range varied from 19 to 88 years, with a median of 52.5 years [13]. In our study, we revised the six classification types of insertion and morphological variants, and we observed no differences.

In an Indian study among 100 patients, the most common pattern of insertion of the uncinate process was type one, accounting for 67.5% (n=135) cases. Type two was the second most common, observed in 18.5% (n=37). Type three attachment was in 9.5% (n=19), and type four was in 4.5%. They observed bilaterally similar attachments in 96%. For the rest of the cases (4%), the attachment patterns varied between sides [14].

In a Taiwanese study [15], they found that a single superior attachment of the UP into the lamina papyracea had the highest prevalence: 70.4%. The second most common type of attachment observed in their study was type two, with a frequency of 10.2%. Different results were reported in other studies with type four (insertion into the lamina papyracea and middle turbinate, 17.5%), type three (insertion into the skull base, 14.4%), and type five (insertion into the lamina papyracea and skull base, 31%), respectively. The distribution of the insertion types of the UP concerning race or ethnicity was statistically significant (p < 0.01). Ethnicity is an important feature to describe and analyze according to Liu et al. [15]. A factor to value that limits is the lack of a standardized classification for the description of attachment sites. This may have been due to the similarity between the classification for the description of attachment sites. There is a need for the development of a standardized classification that can be used in future studies.

In a Turkish study, they also evaluated insertions of the UP to the lamina papyracea (type one/two) were the most common type (62.2%). Type five was the third most common, with a frequency of 14.4%. Landsberg et al. have found types one and two as 70.5% [4]. The difference between these studies may be attributable to racial differences [16].

Upper insertion of the UP into the lamina papyracea has been consistently the most common finding in distinct studies. In our population, UP insertion into the lamina papyracea was observed in 33 to 45% of patients, with no difference observed compared with age group or sex. Interestingly, the insertion into the lamina papyracea of the UP has been associated with patients with frontal sinusitis [3]. These observations are mainly from retrospective studies with a limited number of patients limiting their application.

The patterns of laterality evaluation of the UP have been demonstrated to be similar on the right and left sides [17]. The prevalence of bilateral frontal sinusitis in patients with identical left and right UP insertion is 23%, while its prevalence in non-identical patterns is 14% showing a statistically significant association. Arguments of whether the performance of bilateral surgery in patients with unilateral frontal sinus involvement remains as the UP may play a role in the pathogenesis of frontal sinusitis [17].

With the advancement of new imaging techniques, radiological evaluation with high-resolution CT provides a detailed evaluation of relevant anatomical landmarks for endoscopic sinus surgery.

The limitations of our study include its retrospective design with inherited methodological drawbacks and the limited number of patients in the study. Our study has several methodological strengths that should be considered for future studies. The CT scans consisted of fine-axial slice thickness allowing a detailed evaluation of the bone structures. Additionally, the images were evaluated by head and neck radiology experts.

## Conclusions

The importance of knowledge before performing an endoscopic sinus surgery of the anatomic variations in paranasal sinuses is critical for the surgical team, as well as for the radiologist involved in the analysis of these studies. Anatomical variants of the UP should be considered before any surgical procedure is performed.

Certain anatomical elements of relevance must be verified in every surgical procedure, and, at the same time, it must be noted that even within populations, it is not constant. This entails not only anatomical knowledge as part of the academy for students but also its importance in the development of pathologies and avoiding intraoperative and postoperative complications.
